# Learning and Judgment Can Be Affected by Predisposed Fearfulness in Laying Hens

**DOI:** 10.3389/fvets.2017.00113

**Published:** 2017-07-27

**Authors:** Elske N. de Haas, Caroline Lee, T. Bas Rodenburg

**Affiliations:** ^1^Behavioural Ecology Group, Wageningen University, Wageningen, Netherlands; ^2^Adaptation Physiology Group, Wageningen University, Wageningen, Netherlands; ^3^Commonwealth Scientific and Industrial Research Organisation (CSIRO), Armidale, NSW, Australia

**Keywords:** ambiguity, anxiety, chickens, cognition, fearfulness, judgment, response strategy

## Abstract

High fearfulness could disrupt learning and likely affects judgment in animals, especially when it is part of an animals’ personality, i.e., trait anxiety. Here, we tested whether high fearfulness affects discrimination learning and judgment bias (JB) in laying hens. Based on the response to an open field at 5 weeks of age, birds were categorized as fearful (FC) by showing no walking or vocalizing or non-fearful (NFC) by showing walking and vocalizing. At adult age, birds (*n* = 24) were trained in a go–go task to discriminate two cues (white or black) with a small or large reward. Birds that reached training criteria were exposed to three unrewarded ambiguous cues (25, 50, and 75% black) to assess JB. Task acquisition took longer for FC birds than for NFC birds, due to a left side bias, and more sessions were needed to unlearn this side bias. Changes in trial setup increased response latencies for FC birds but not for NFC birds. A larger number of FC birds than NFC birds chose optimistically in the last ambiguous trial (25% black). FC birds had a longer latency to choose in the ambiguous trial (75% black) compared to NFC birds. Prior choice in ambiguous trials and a preceding large or small trial affected latencies and choices for both types of birds. Our study showed that fearfulness was associated with differences in discrimination learning ability and JB. It appeared that FC birds used a rigid response strategy during early learning phases by choosing a specific side repeatedly irrespective of success. FC birds were more affected by changes in the setup of the trials in comparison to NFC birds. We speculate that FC birds are more sensitive to changes in environmental cues and reward expectancy. These factors could explain how high fearfulness affects learning.

## Introduction

Discrimination tasks are well-used cognitive tasks to assess learning in humans and animals (1–4). In these tasks, cues are accompanied by different types of outcomes (reward, punishment, or social stimulus) (5). The level of acquisition of these associations is measured by the number of correct runs and the number of trials to reach a training criterion. These measurements are interpreted as a subject’s cognitive performance to associate in a specific task (5). Cognition entails the ability to process and acquire new information and learn associations and to store this information, memorize it, and use it in future decision-making (6). A subject’s fearfulness may influence its learning style and strategy. As a trait, fearfulness identifies a feature of an animal’s personality (7) reflecting how an animal interacts with its social and physical environment (8, 9) making fearfulness likely to play a role in cognition and judgment. By having a highly fearful predisposition, i.e., a tendency to develop anxiety, animals may give more attention to certain cues over others (10), thereby being biased to negative or threatening cues (11). Trait anxiety can lead to selective information-processing (12), disrupted motivation (13), and attention, memory, and judgment bias (JB) (11, 14). JB has been shown to play a large role in an animal’s expectation of a certain outcome (15). JB refers to interpreting an ambiguous cue to predict as having a positive or negative outcome, thereby reflecting pessimistic or optimistic tendencies (16), which can be affected by anxiety (17). Highly anxious humans, for example, interpret ambiguous stimuli as threatening compared to humans with low anxiety (18, 19). Likewise, young laying hen chicks with high anxiety are more inhibited to approach an ambiguous stimulus compared to chicks with low anxiety (20). It is unknown whether JB exists in adult chickens with a fearful predisposition. Earlier, we found that in adult laying hens, low learning success correlated with high fear and stress levels (21). It is unknown whether differences in learning relate to predisposed high fearfulness assessed at a young age.

Here, we aimed to answer the question, whether having a fearful predisposition affects discrimination learning and JB in laying hens. To answer this question we assessed responses of laying hen chicks for 5 min in a novel open field (OF) test at 5 weeks of age—a validated measurement for anxiety in many species (22) including chickens (23). In young chicks, short exposure (minutes) to the OF test facilitates behavior indicative for anxiety tendencies while long exposure (hours) reflects the tendency for a more depressive-like state (23). The OF has been validated with anxiolytics. For example, anxiety-induced suppression of activity in the OF is abolished after treatment with an anxiolytic (24), which also affects JB (25). When isolated, young chicks (up to ~10 days of age) aim to social reinstate by high-pitched vocalizations (distress or alarm calls) (26). When slightly older (i.e., 4–5 weeks of age) chicks respond to threatening situations, like exposure to a novel arena or stimuli, by becoming passive to reduce detection by a predator or highly active seeking to escape and social reinstate. Passive responses/low levels of activity in the OF indicate high fear (27) and are generally interpreted as reflecting predisposed high anxiety (20). Activity in the OF in laying hen chicks of 4–5 weeks of age is regulated by 10 quantitative trait loci in chickens, identical to OF behavior of mice and human mental disorders associated with anxiety (28). Our chicks which responded passively to exposure in the OF at 5 weeks of age also tended to have longer duration of tonic immobility (TI) at an adult age (29). These studies support that a low level of activity in the OF when young reflects a fearful predisposition of an animal. Here, we assessed differences of birds characterized as fearful or non-fearful in cognitive performance and JB in a two-choice discrimination task at adult age. We also assessed if our characterization of fearfulness is reflected by their response to a novel environment/social isolation test at 1 week of age, as executed earlier in chickens to assess separation anxiety (30).

## Materials and Methods

This experiment was approved by the Animal Care and Use Committee of Wageningen University & Research (the Netherlands) in accordance with Dutch legislation on the treatment of experimental animals the ETS123 (Council of Europe 1985) and the 86/609/EEC Directive. This experiment was conducted from October 2012 until July 2013. 217 chicks were tested for FC and NFC characterization for a larger experiment (29). From these, we retained 24 birds for this analysis based on most contrasting OF behavior and without feather damage so as to assure that birds were not fearful as a consequence of being feather pecked.

### Housing Conditions from 1 to 5 Weeks of Age

White leghorn laying hybrid chicks (Dekalb White) arrived from the hatchery at 1 day of age. Chicks were housed in a communal pen until 5 weeks of age. The communal pen measured 8 m × 8 m with wire walls of 2.5 m height. Chicks had *ad libitum* access to mashed food (commercial pullet starter 1 diet) from five rectangular feeders and water from three round water towers in addition to 25 water nipples which were supplied evenly throughout the pen. Artificial fluorescent light was provided. In the first week of life, light was on for 4 h followed by a 4 h dark period. From day 8 of age onward light was provided for 8 h per day, where at the end of every week 1 h was added until 15 h light per day were reached. This setting was maintained throughout the experiment. Temperature was gradually decreased from 33°C by lowering the temperature by 1°C weekly until 19°C was reached at 10 weeks of age. Pens were supplied with wood shavings on the floor and from day 21 of age wooden rectangular perches were provided. At 1 week of age chicks were weighed individually and marked with a plastic label through the skin of the neck which had a specific number/color combination to enable bird ID.

### Housing Conditions from 5 Weeks of Age Onward

From 5 weeks of age onward, chicks were housed in groups of eight. Pens (2 m × 1 m) consisted of a floor area with wood shavings, an elevated wooden perch (at 50 cm height), a rectangular feeding trough [50 cm (*l*)] at 15 cm height on one side of the pen, and a drinking line with five water nipples at 20 cm height on the other side of the pen. Birds had *ad libitum* access to pelleted food (commercial layer diet from Agruniek Rijnvallei, the Netherlands) and water and received scattered grain and straw on the floor each day around 8:00 a.m. The inner walls between pens had a cardboard cover of 50 cm high to prevent visual contact with birds from adjacent pens.

### Behavioral Observations

#### Novel Environment Test

At 1 week of age, chicks were individually subjected to a novel environment/social isolation test (NET). We chose to incorporate this test, so as to assess repeatability of responses in a similar kind of anxiety test as the OF. Chicks were placed in a white plastic bucket with a diameter of 40 cm and of 50 cm height. Latency to walk, latency to vocalize, and number of vocalizations (distress calls) were recorded. Behavior was scored for 60 s by direct observation *via* a video camera by an observer out-of-sight of the test subject.

#### Open Field Test

**Figure 1:**
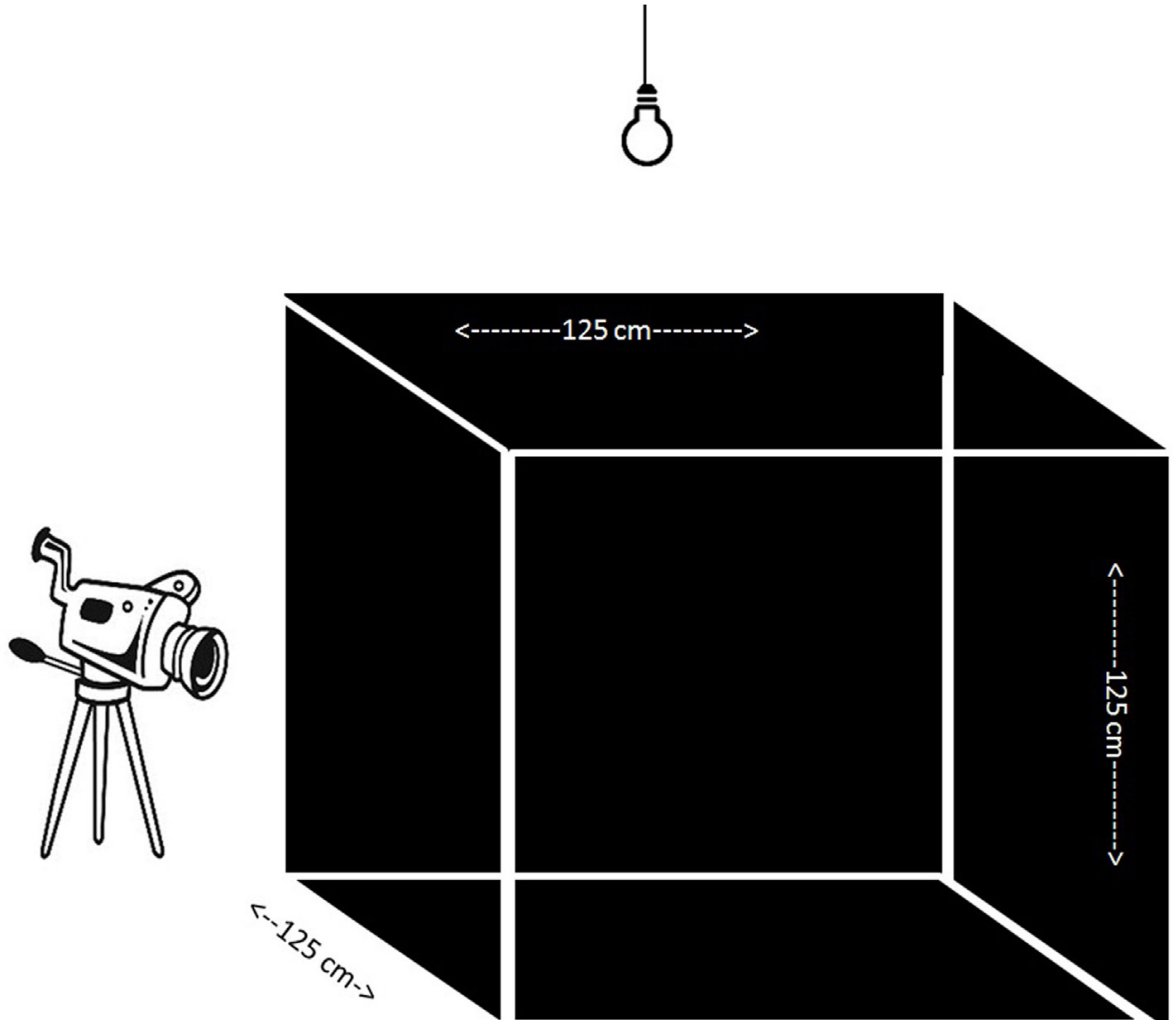
Open field test apparatus containing three black walls and one transparent through which camera recording took place. An extra light bulb enabled evenly distributed light in the apparatus.

At 5 weeks of age, chicks were individually subjected to an open field (OF) test (Figure 1). The OF was a wooden square construction of 125 cm × 125 cm × 125 cm with three black walls, one transparent wall through which video recordings were obtained, and a black floor with white tape to separate four evenly distributed squares of the floor surface and a wire mesh on top of the OF. A 90-W light bulb was placed 50 cm above the OF, in addition to the fluorescent light of the room, which ensured an equal distribution of light (±25 LUX) over the test apparatus (measured with a Voltcraft MS-1300 LUX device, Conrad, Oldenzaal, the Netherlands). Chicks were randomly chosen and individually caught from the communal pen and brought to the testing room in a cardboard box. In the testing room, a chick was placed in the middle of the OF which was kept dark until the start of the test. Behavior was scored for 5 min by one observer from live video recordings (tunnel security camera with external recorder) in an adjacent room using Observer XT 7.0.2 (Noldus, Wageningen, the Netherlands). The latency to walk, the latency to vocalize, and the number of vocalizations were recorded. For the characterization we focused on latency to walk and/or vocalize to characterize chicks. A fearful chicken (FC) was characterized by a latency longer than 300 s to walk and vocalize, reflecting a freezing non-vocalizing chicken. A non-fearful bird (NFC) was characterized by vocalizing before 30 s and walking before 200 s to create a stringent contrast with FC birds. From the total population we chose the 24 most extreme cases representing FC and NFC birds.

#### Go–Go Two Choice Visual Discrimination Task

From 35–42 weeks of age, 24 hens were trained in a go–go discrimination task. Hens were required to make a choice whereby we could assess their discrimination abilities. Here, they had to learn to associate two different combinations of cue types associated with a different food reward size. As a form of Pavlovian conditioning (5), hens thus needed to learn the association between two background colors and two symbols. This test was designed by Hernandez et al. (31), adapted for chickens based on starling research by Bateson and Matheson (32). Each training week was defined by four consecutive days of training. A training day consisted of 10 consecutive trials per bird. A maximum of 28 training days were performed. On training day 29 a JB test was executed. The training and testing took place in a sound-dampened test room, by live observations supported by camera recordings. Two trainers executed the training. One trainer always handled the birds and observed the behavior while the other trainer always handled the apparatus and cue cards.

##### Test Apparatus

**Figure 2:**
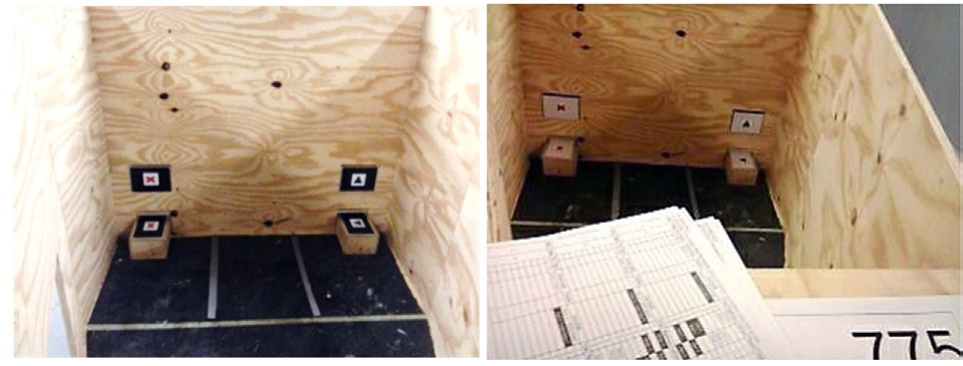
Inside the test apparatus. Cues are represented on feeder lids and metal cue cards above feeders. Decision lines are represented by gray scotch tape markings on the floor of the apparatus. White cue cards indicate small reward trial, and black cue cards indicate large reward trials. Papers represent location from which observations took place and number is hen id.

A wooden test apparatus with a semitriangular shape was used [identical to Ref. (21)]. Walls were of 50 cm height. The floor had a black surface (*l* × *w* = 200 cm × 120 cm). The apparatus contained a separate start box (*l* × *w* = 60 cm × 25 cm) with a wooden guillotine door. At the opposite of the start box, two feeders (8 cm × 12 cm × 15 cm) were exposed through slots (Figure 2). On top of the feeders, metal lids (*l* × *w* = 11.8 cm × 26 cm) with plasticized cues (see below) were presented. Lids could be removed separately from the feeders providing birds access to the food rewards. Ten cm above the feeders, through a narrow slot (*l* × *w* = 1 cm × 17 cm), rectangular metal cue cards with plasticized cues (*l* × *w* = 11 cm × 16 cm) were presented on which the same cue was shown as on the lid of the corresponding feeder. Visual markings (with gray scotch tape) around the feeders on the floor (*l* × *w* = 40 cm × 50 cm) indicated decision lines. A choice for one of the feeders was recorded when both legs and feet of the bird crossed a decision line.

##### Cues

**Figure 3:**
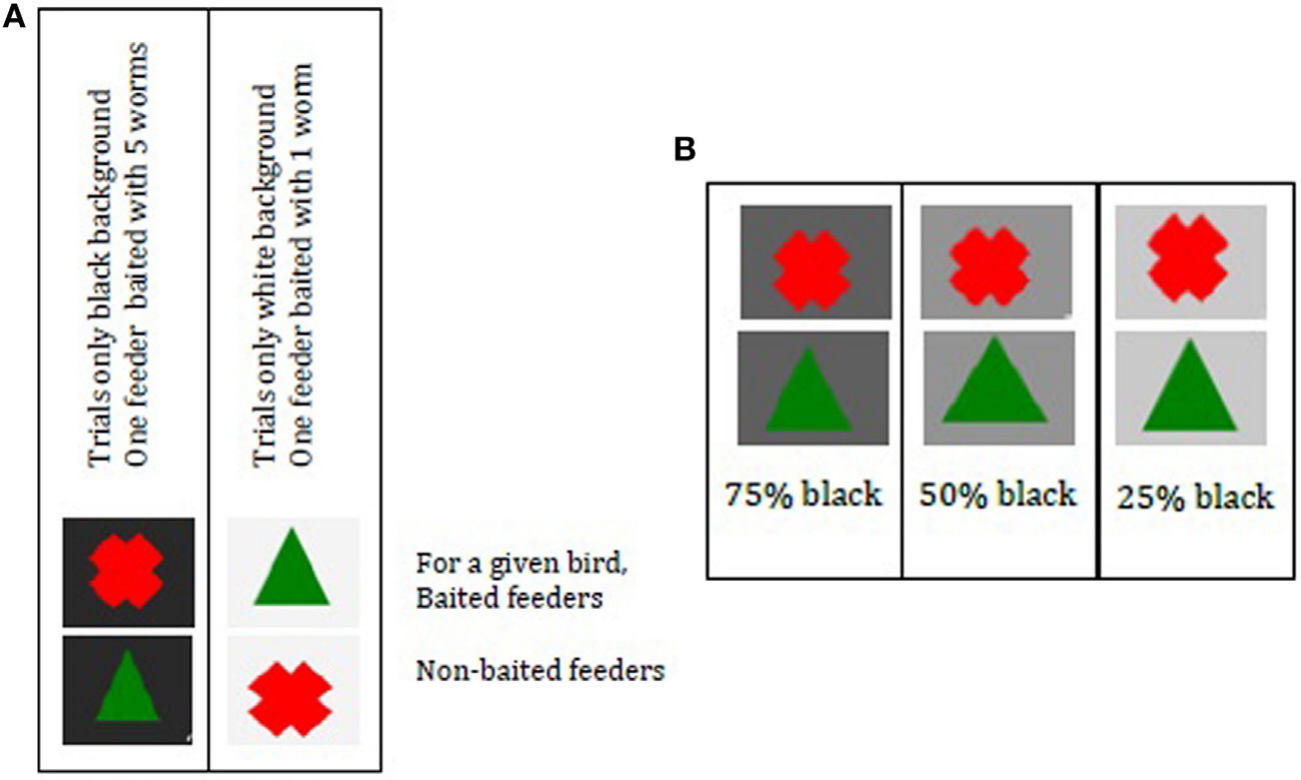
**(A)** Cue cards to indicate reward size and symbols to indicate baited feeder. Per trial one color was used where one feeder was baited. The background color indicated reward size, black indicated the possibility to find five worms, and white indicated the possibility to find one worm. **(B)** Cue cards with ambiguous background color 75, 50, and 25% black. Per trial, two feeders were present with only one background color. Choice for a symbol was associated with predicting a large or small reward **(A)**. Ambiguous trials were unrewarded. *Note*: if a bird would have a reward under cross black and triangle white [as in **(A)**], her choice for cross under gray would represent an optimistic bias. Her choice for triangle would represent a pessimistic bias.

Birds needed to learn under which symbol (green Δ or red X) a food reward could be found. Symbols were shown either on a black or on a white background (Figure 3A). Background color indicated size of food reward; five mealworms were associated with black (RGB 248 0% transparency) and one mealworm was associated with white (RGB 248 95% transparency). Trials always contained one background color (i.e., L trials = large reward: black background; S trials = small reward: white background), which was fixed for all birds. The training of large and small rewards was to allow the birds to associate one symbol with reward and the other with no reward in a “small reward situation” (white background) and then the symbols had the opposite meaning for the large rewards (black background).

One feeder was baited per trial. For a given bird, black + red X was five worms, while black + green Δ had no reward. For that same bird, white + green Δ was one worm and white + red X had no worm (see Figure 3A). Reward–cue combinations were balanced across FC and NFC.

Birds were exposed to ambiguous cues in the JB test at day 29. These had gray-toned backgrounds, 25, 50, or 75% black (RGB) [Figure 3B; adapted from Ref. (31)]. If birds were trained that the black background yielded a large reward when the green Δ is chosen, then the white background yielded a small reward when the red X was chosen and vice versa. Consequently, a choice for the green Δ during the ambiguous trials indicated that the bird was interpreting the gray background to be more similar to black than white—an “optimistic” response anticipating the larger reward.

##### Training

The training period consisted of four training phases: phase 1: acquisition; phase 2: S trials; phase 3: one L trial; and phase 4: consolidation of L + S cues (three L trials). Training was identical as described by Ref. (21, 31) with the exception of phase 2. Furthermore, the training criteria were set to 80% correct choices over two consecutive days. See Table 1 for details of training setup and sample sizes per period.

**Table 1:** Description of training phases for judgment bias in fearful and non-fearful characterized chickens.

Phase	Days	Cues^a^	Order^b^	Side baited feeder^c^	Switch options^d^	Objective	Sample size	
							Non-fearful birds	Fearful birds
Habituation	1–5	No	None	Random	NA	Familiarization with test arena and cues in pairs	12	12
Phase 1: acquisition	6–10	S and L	None	Random	All	Acquisition of S cues	12	12
Phase 2: S trials	11–16	S	None	Preset	First 3	Undo side-bias	12	12
Phase 3: 1 L trial	17–20	S and L	None	Random	No	Learn L cue while maintaining S cues	10	8
Phase 4: 3 L trials	21–28	S and L	Large reward trials: 4, 7, and 9; 2, 5, and 8; and 1, 2, and 8	Random	No	Solidify association of S and L cues	10	8
Judgment bias	29	S, L, and A	Trials 1–9: L, S, 50%; L, S; 75%; and L, S, 25% or S, L, 75%; S, L, 50%; and S, L, 25%	Semirandom	No	Assess judgment bias	10	8

*^a^Cues: S = small reward cues displayed (one mealworm), L = large reward cues displayed (five mealworms), and A = ambiguous cues (no mealworms)*.

*^b^Order: trials where specific cues were displayed, none indicated no specific order of trials*.

*^c^Side-baited feeder: random was 50/50 left and right, preset: meant baited feeder opposite to biased side in a stepwise increasing number from 5 to 10, semirandom: assigned to preceding small or large reward prior to ambiguous choice*.

*^d^Switch options: opportunity to switch when an incorrect choice was made. NA = not applicable, No = no switch after incorrect choice, first three: switching only in first three trials*.

*Training days contained sessions of 10 trials per day*.

##### Habituation

Birds were habituated in their home pen to feeders and mealworms on days 1 and 2. We recorded whether birds ate from the feeders in the home pen. From day 3 onward, birds were habituated in the test apparatus in pairs of similar characterization from the same pen (both FC and NFC). This was conducted to limit isolation stress for the test subject. On day 3, hens had access to the whole apparatus with open feeders filled with worms. We recorded whether birds ate from the feeders in the test apparatus. On day 4, birds started from the start box with the guillotine door open. Once the worms were consumed from the feeders birds were caught and placed in the start box again, and a consecutive trial was executed with refilled feeders. This setup was repeated on days 5 and 6.

##### Phase 1: Acquisition Phase

From day 7 onward data collection took place on an individual level. Cue cards on lids and walls were both present. The acquisition phase contained four sessions with five S and five L trials. The location of the baited feeder was always randomized for both S and L trials. This setup was used as we wanted birds to focus on the combination of background colour and symbol rather than on location of the baited feeder. Birds were allowed to switch between feeders following an initial incorrect choice.

##### Phase 2: Adapted Phase

During phase 2 only S trials were given. In phase 1, we noticed that 70% of the birds repeatedly chose the feeder on one side irrespective of finding a reward (i.e. more than 7 out of 10 trials). We calculated birds’ side choices when no reward was obtained by subtracting the number of side choices from the number of rewards given on that particular side. A more negative number indicated a side bias (i.e., going to left but not finding a reward). In our previous study (21), side bias severely hampered learning; therefore, birds with a side bias were subjected to an adapted training scheme. We chose to adapt the training so as to increase the number of individuals learning the task. From day 11 till 16 the rewarded feeder was opposite to the preferred side. Birds that did not develop a side bias remained on a randomized scheme with S trials. We increased the number of times the baited feeder was on the non-preferred side, stepwise from 5 to 10 trials per session. Birds could switch between feeders in the first three trials, hereafter wrong choices remained unrewarded. The criterion to move to the next phase was 80% correct S trials on randomized choices given on two consecutive days *(criterion phase 3)*.

##### Phase 3: 1 Large Reward Trial Phase

On day 17 implementation of one L trial was initiated. Four sessions containing one L trial and nine S trials were used. We chose one L trial to slowly increase the complexity of the test, aiming to maintain learning performance for most birds. The criterion to move to the next phase was 80% accuracy to S trials and 100% to the L trial on two consecutive days (*criterion phase 4*).

##### Phase 4: Consolidation Phase

On days 21–28, birds were subjected to seven S and three L trials per session. These sessions had predefined L trials, similar for all birds, in the following recurring order: trials 4, 7, and 9; trials 2, 5, and 8; and trials 1, 2, and 8. We chose to use three L trials vs. seven S trials to maintain the motivation of birds for the S trials and not create omission in S trials because of preference for the large reward. We recorded a shorter latency to choose in L trials than in S trials (data not shown). The criterion for the JB test (*criterion JB test*) was reached if birds achieved a learning score of 80% of both L and S trials on two consecutive days.

### JB Task

The JB test consisted of nine trials. In three of those trials ambiguous cues were presented of different % of RGB: 25, 50, and 75% black background (see Figure 3B). The test also included three L trials with black background and three S trials with white background. When a hen approached the symbol associated with the large reward (black), her choice would be interpreted as optimistic. When a hen approached the symbol associated with the small reward (white), her choice would be interpreted as pessimistic (33). The trial preceding the ambiguous trial can affect latency to choose (31). Therefore, two trial sequences were used, preceding small trials (L, S, 50% gray; L, S, 75% gray; and L, S, 25% gray) or preceding large trials (S, L, 75% gray; S, L, 50% gray; and S, L, 25% gray). We chose to use unrewarded ambiguous cues and expose birds once to each of these cues once. Rewarded or unrewarded ambiguous trials can affect consecutive choices in ambiguous trials (14). However, we wanted to compare with earlier studies with the same setup (21, 31).

### Statistical Analysis

Data were analyzed by SAS 9.3 (SAS, Cary, NC, USA). Variables were checked for normal distribution of residuals based on a general linear model (GLM) with a fixed effect of characterization (FC or NFC) and the error term of characterization nested within pen to correct for animals from the same pen. Because FC birds all had the maximum latency to walk and vocalize in the OF we could not conduct a linear regression analysis. Therefore, instead, models with a fixed effect of characterization were applied. The GLM was used to test the effect of characterization on the latency to walk in the NET, number of sessions to obtain the different training criteria, and latency to choose in the JB test. Latencies to choose on days 17 (one L) and 21 (three L trials) were tested with a *t*-test for group comparison. Spearman correlations were calculated between latency to walk in the NET and OF. Repeated measurements of latency to choose, learning score (representing the percentage of correct choices based on calculation of errors), and side bias (representing the number of choices to a particular side corrected for baited side) were analyzed with a MIXED model. Fixed effects were characterization, with the repeated effect of time and the interaction between characterization and time. The random effect included pen. The model was run by phase to check for differences in performance between phases. A binominal logit link GENMOD model tested effects of characterization on binominal variables: number of birds that developed side bias (yes/no), number of birds that met the training criteria (yes/no), and number of birds that chose optimistically in ambiguous trials (yes/no). To test if choice in the last ambiguous trial (25% black) was affected by choice in the preceding ambiguous trials we added this to the GENMOD model as a fixed factor. In the JB test, we tested if having an L or S trial prior to the ambiguous trial affected the latency to choose by adding preceding L or S trial as a fixed effect in the GLM. Data are expressed as mean ± SEM with *P* smaller than 0.05 classified as statistically significant.

## Results

### Behavioral Response to the Fear Tests

In the NET at 1 week of age, FC birds tended to have a longer latency to walk compared to NFC birds (56.8 ± 2.2 vs. 45.2 ± 5.0 s, *F*_1,21_ = 3.83, *P* = 0.06). No differences in vocalization responses between NFC and FC birds were found in the NET (latency: 13 ± 4 vs. 10.7 ± 5 s, *F*_1,21_ = 0.09, *P* = 0.76 and vocalizations: 37.1 ± 9 vs. 55.4 ± 8 s, *F*_1,21_ = 1.09, *P* = 0.17). In the OF at 5 weeks of age, none of the FC birds walked or vocalised within the test duration of 300 s. NFC birds had an average latency to walk of 134.5 ± 12.8 s (max: 147 s, mean: 103 s, and min: 4 s) and average latency to vocalize of 20.9 ± 6.3 s (max: 71.4 s, mean: 10.5, and min: 0.4 s). Latency to walk in the NET and OF was positively correlated (*r* = 0.73, *P* = 0.05). Latency to vocalize or vocalizations in the NET and OF were not correlated, also not with walking latency (for all combinations: *r* > 0.15, *P* > 0.35).

### Training Sessions to Criteria

Eighteen out of 24 birds learned the task, 10 NFC birds (80%) and 8 FC birds (70%) (see Table 2). FC birds needed more sessions to reach the *criterion of phase 3* (16.1 ± 1.2 vs. 12.8 ± 1.0, *F*_1,20_ = 5.27, *P* = 0.03) and the *criterion of phase 4* (19 ± 1.2 vs. 16.2 ± 1.0, *F*_1,18_ = 6.10, *P* = 0.03). No significant difference existed in the number of sessions to reach the *final criterion* between FC and NFC birds (25.5 + 1.1 vs. 23.2 + 1.3, *F*_1,18_ = 2.23, *P* = 0.15). The number of sessions needed to unlearn side biases tended to be higher for FC birds than for NFC birds (5.7 + 0.3 vs. 3.14 + 1.0, *F*_1,18_ = 4.21, *P* = 0.06). A larger proportion of the FC birds needed adjusted training compared to NFC birds (92 vs. 50%, χ^2^ = 6.02, *P* = 0.01).

**Table 2:** The number of fearful and non-fearful characterized birds able to reach criteria in a visual association task.

	Fearful characterized birds	Non-fearful characterized birds	Statistical test and *P*-value
**Number of sessions needed to reach training criteria^a^**			
Reach criterion phase 3 (1 L trial)	**16.1 ± 1.2**	**12.8 ± 1.0**	***F*_1,20_ = 5.27, *P* = 0.03**
Reach criterion phase 4 (3 L trials)	**19.75 ± 1.2**	**16.2 ± 1.0**	***F*_1,17_ = 6.10, *P* = 0.03**
Reach final criterion (judgment bias test)	25.5 ± 1.1	23.8 ± 0.9	*F*_1,17_ = 02.23, *P* = 0.15
Unlearn side-bias	**5.70 ± 0.3**	**3.14 ± 0.8**	***F*_1,17_ = 4.21, *P* = 0.06**
**Number of birds enabled to reach**			
Final criteria	8 out of 12 (70%)	10 out of 12 (80%)	χ^2^ = 0.89, *P* = 0.35
**Number of birds needed**			
Adjusted training	**11 out of 12 (92%)**	**6 out of 12 (50%)**	**χ^2^ = 6.02, *P* = 0.01**
For which adjusted training was successful	8 out of 11 (75%)	4 out of 6(80%)	χ^2^ = 0.09, *P* = 0.75

**Figure 4:**
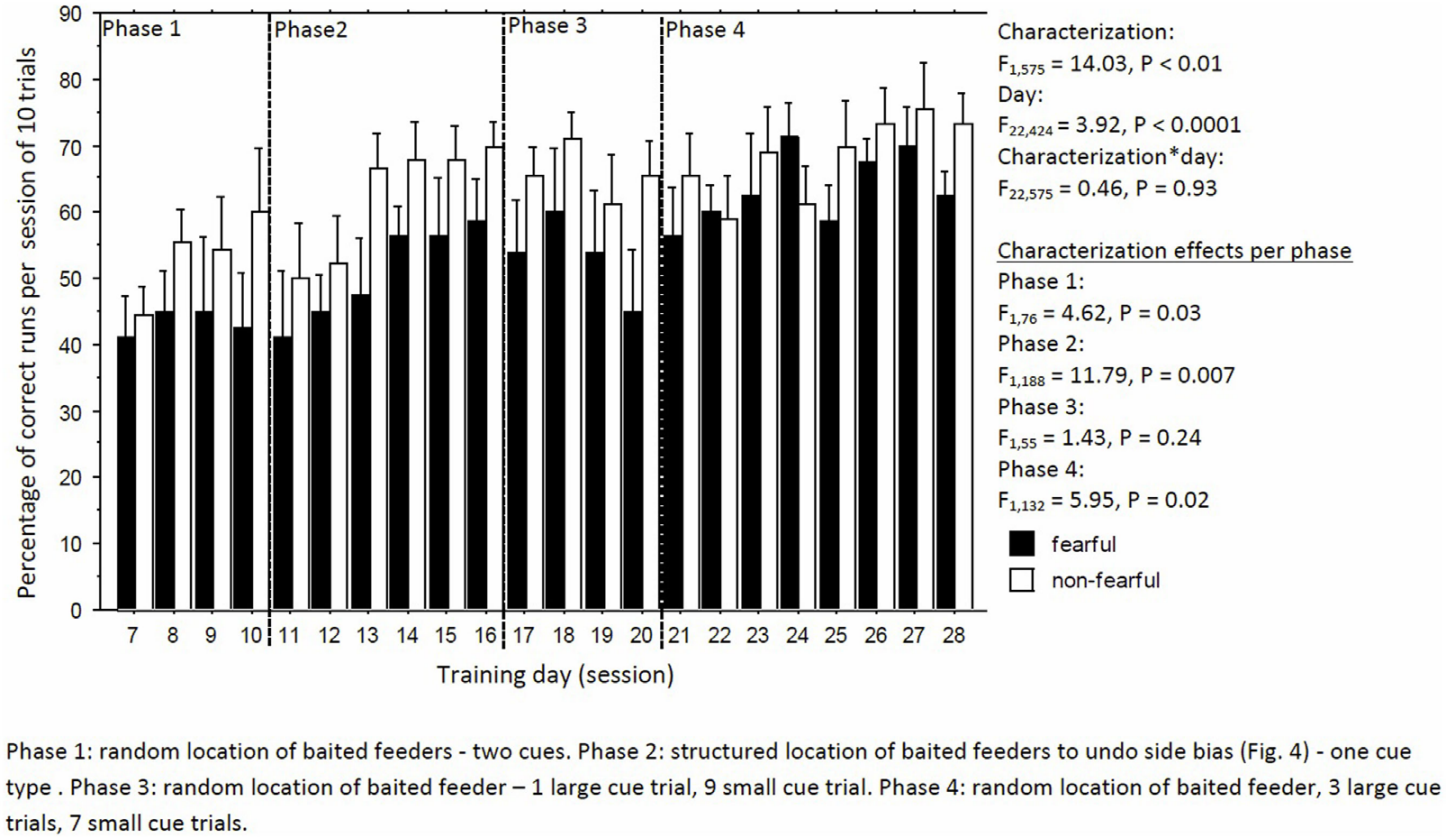
The percentage of correct runs per trial of 10 runs of fearful and non-fearful characterized birds.

*^a^Criteria 80% on last two consecutive trials. Phase 3 criterion led to sessions of one large reward trail and nine small reward trials. Phase 4 criterion led to sessions of three large reward trials and seven small reward trials. Final criterion led to the judgment bias test of three ambiguous trials within three small reward and three large reward trials. Adjusted training was added to chickens which had developed a side bias (Figure 4)*.

*Numbers in bold indicate P < 0.05*.

### Learning Performance, Side Choices, and Side Bias

**Figure 5:**
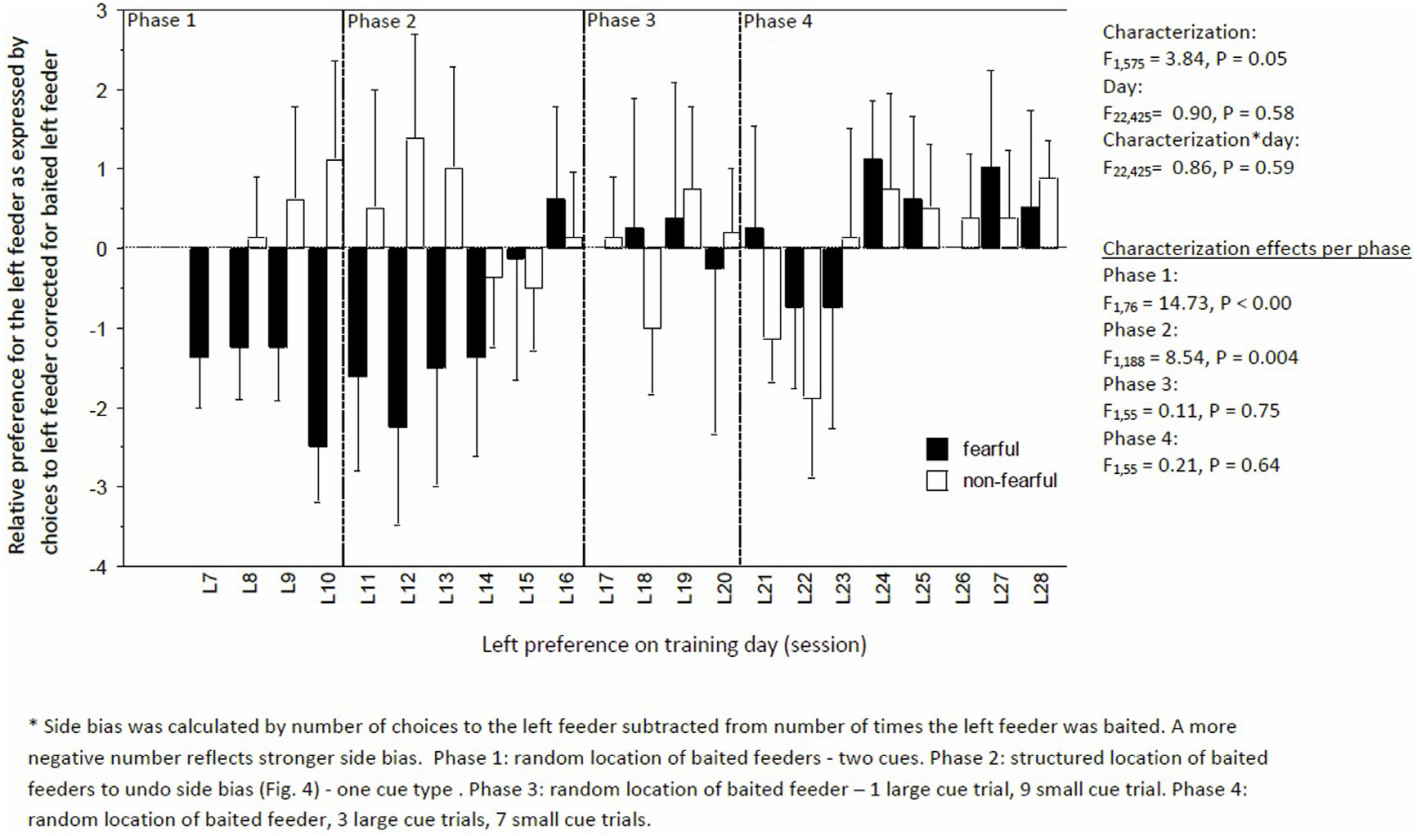
Side bias to the left feeder corrected for the times the feeder was baited of fearful and non-fearful characterized birds. A negative number indicated a stronger bias to the left.

Learning performance fluctuated but increased over time, irrespective of characterization (day: *F*_22,424_ = 3.92, *P* < 0.0001; day × characterization, *F*_22,575_ = 0.46, *P* = 0.93, Figure 4). But, FC birds consistently had a lower learning performance than NFC birds (*F*_1,575_ = 14.03, *P* < 0.01; Figure 4). Specifically, FC birds were significantly worse than NFC birds during the acquisition phase (phase 1: percentage of correct runs 45.4 ± 2.9 vs. 54.6 ± 2.8%, *F*_1,76_ = 4.62, *P* = 0.03), in the adapted learning phase (phase 2: 51.2 ± 3.5 vs. 62.0 ± 3.2%, *F*_1,188_ = 11.79, *P* = 0.007), and the final learning phase (phase 4: 60.5 ± 2.9 vs. 68.2 ± 2.8%, *F*_1,132_ = 5.95, *P* = 0.02). No differences in learning performance were found in phase 3 (56.8 ± 5.8 vs. 61.8 ± 5.6%, *F*_1,55_ = 1.43, *P* = 0.24). Overall, FC birds had a stronger side bias toward the left side than NFC birds, with significance of *P* < 0.05 in phases 1 and 2 (Figure 5).

### Latency to Make a Choice

**Figure 6:**
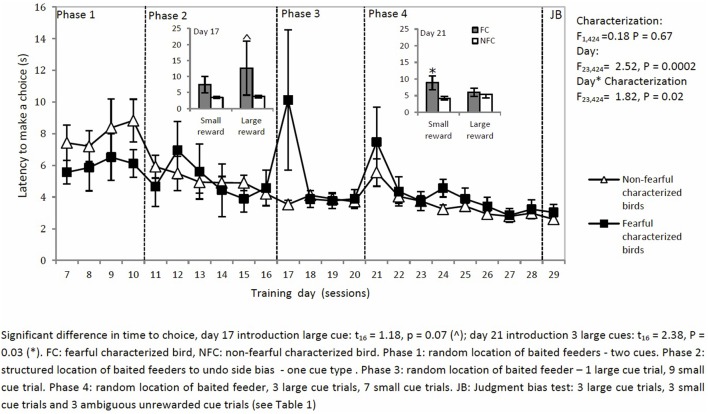
The latency to make a choice in a two-choice discrimination task for fearful and non-fearful characterized birds.

The latency to make a choice decreased over sessions for both types of birds except on two specific days where the setup of trials was changed (characterization: *F*_1,424_ = 0.18, *P* = 0.67: day × characterization: *F*_22,424_ = 1.82, *P* = 0.02: Figure 6). On days 17 and 21, latency to choose was affected by characterization. On day 17, introduction of an L trial led to a longer latency to choose in the L trial for FC birds than for NFC birds (*t*_16_ = 1.18, *P* = 0.00). On day 21, introduction of three L trials led to a longer latency to choose in the S trials for FC but not for NFC birds (*t*_16_ = 2.38, *P* = 0.03).

### JB Test

#### Choices in the JB Test

In the JB test, nine trials were given. Per trial birds could choose between a feeder with a triangle or cross. Three trials had a black background on the feeders (five worms to be found under either a triangle or cross), three trials had a white background on the feeders (one worm to be found either under triangle or cross), and three trials had a gray background on the feeder (25, 50, and 75% of black) with no worm to be found under triangle or cross. If in the gray trials a bird chose for a symbol predicting five worms this would indicate optimistic bias. The last gray trial was 25% black. We found most effects on this last ambiguous trial. This was more often chosen as predicting a large reward in FC birds compared to NFC birds (Table 3; Wald χ^2^ = 4.22, *P* = 0.04). This indicated that FC birds judged this background to predict a large reward resembling an “optimistic” judgment. The choice in the 25% black ambiguous trial was affected by the choice in the 75% black ambiguous trial (Wald χ^2^ = 4.33, *P* = 0.04). If FC birds had chosen for 75% black reflecting the S reward, they chose more often than NFC birds for 25% black reflecting the L reward—this was the opposite for NFC birds (Table 3). The choice of the 50% black or 75% black ambiguous cue was not affected by characterization (χ^2^ = 0.08, *P* = 0.77; χ^2^ = 1.43, *P* = 0.24, Table 3).

**Table 3:** Choices and time to choose for a specific feeder in the judgment bias task for fearful and non-fearful characterized birds.

	Fearful characterized birds	Non-fearful characterized birds	Statistical test and *P*-value
**Number of birds choosing the ambiguous as representing the large reward**			
25% black	**6 out of 8**	**2 out of 10**	χ^2^ = 4.22, *P* = 0.04
50% black	3 out of 8	5 out of 10	χ^2^ = 0.08, *P* = 0.77
75% black	3 out of 8	6 out of 10	χ^2^ = 1.43, *P* = 0.24
**Time to choose (s) during the judgment bias test**			
0% RGB—small reward trial	3.4 ± 0.4	3.0 ± 0.4	*F*_1,15_ = 0.60, *P* = 0.45
25% RGB—non-rewarded ambiguous trial	3.0 ± 0.3	2.7 ± 0.4	*F*_1,15_ = 0.37, *P* = 0.55
50% RGB—non-rewarded ambiguous trial	2.6 ± 0.3	2.4 ± 0.17	*F*_1,15_ = 0.24, *P* = 0.63
75% RGB—non-rewarded ambiguous trial	**2.7 ± 0.3**	**2.0 ± 0.3**	*F*_1,15_ = 3.45, *P* = 0.08
100% RGB—large reward trial	2.6 ± 0.3	2.3 ± 0.2	*F*_1,15_ = 1.35, *P* = 0.26
**Switch choice in ambiguous trials 1–3**			
From large to small	**0 out of 8**	**7 out of 10**	χ^2^ = 4.33, *P* = 0.04
From small to large	**6 out of 8**	**0 out of 10**	

*Numbers in bold indicate P < 0.05*.

#### Latency to Choose in the Judgment Bias Test

Latency to choose in the 75% ambiguous trial tended to be longer for FC birds than for NFC birds (3.54 ± 0.48 vs. 2.49 ± 0.22 s, *F*_1,15_ = 3.45, *P* = 0.08). Latency to choose in the S, L and 25 and 50% ambiguous trial was not affected by characterization (Table 3). A preceding L trial increased latency to choose in the 50% ambiguous trial (β 0.8 s: *F*_1,17_ = 25.7, *P* = 0.02).

## Discussion

We characterized laying hen chicks at 5 weeks of age as fearful (FC) or non-fearful (NFC) on the basis of their freezing response in the OF. As adults, hens were exposed to a discrimination task with two cue types (white, black) and associated reward sizes; small (one worm) or large (five worms), respectively. Seventy percent of the birds learned the task, but of these 75% needed extensive adjusted training. Compared to NFC birds, FC birds had a lower cognitive performance, needed more trials to reach the training criterion, and needed more sessions of adapted training. Changes in trial setup increased response latencies for FC birds but not for NFC birds. These results indicate that predisposed fearfulness negatively affects learning in chickens.

In support of our characterization based on OF behavior, latency to walk in OF and NET were positively correlated. In both tests, an animal is exposed—post-handling—to a brightly lit novel environment while being in social isolation. Despite the lack of an actual imminent threat, animals which freeze, i.e., not walk, are assumed to perceive this situation as threatening (33). Walking in the OF can also reflect exploration, novelty seeking, escape behavior, and can relate to impulsivity (34). The freezing response seen in FC birds, however, more likely reflects an adaptive mechanism which reduces detection by predators, as a form of passive coping and excessive fear. As mentioned in the introduction, the response of chickens in the OF is repeatable (28) and heritable (35), but in mice the response strength declines due to habituation when repeated multiple times (36). Although the OF was conducted once, our characterization of FC birds was supported by a longer TI in FC birds vs. NFC birds at juvenile and adult age (29) and by a longer latency to walk in the NET. In many species, freezing in the OF is indicative of anxiety (22). Anxiety has been associated with alterations in learning in other species (37, 38) and pessimistic JB in young chicks (20). FC birds had a lower learning performance and needed more trials to learn the task than NFC birds. These results are comparable to those found in chickens which were fearful of a novel object and did not pass the criteria for a JB test (39). However in mice, an increase in proportion of time spent in the middle of the OF correlated positively with more efficient learning of a specific test based on operant approaching, discrimination, and on other cognitive domains (40).

FC birds were less successful in learning and performed more left side choices (i.e., side bias) compared to NFC birds. Hens with high fear and stress levels can develop a side bias as we showed earlier (21). In rats which froze in the OF more side errors were made in a Y-maze task (41) and in a water maze task (38) based on place recognition memory. These rats exhibited a stimulus–response strategy which linked to inherent high trait anxiety (41). Our result on side bias in chickens here and that in Ref. (21) direct to a stimulus–response learning strategy adapted by FC hens. Stimulus–response learning strategies are specific motor actions in a repeating sequence or to always turn left or right to reach a goal (41). These responses “rely on cues that signal a specific sequence toward a goal, or upon a discrete cue proximal to a goal that signals its location” (41). These responses are known to be rigid and less adaptive to changing conditions (42). FC birds also needed more sessions to unlearn their side bias compared to NFC birds. In the adaptive learning phase, we reinforced birds’ incorrect choices so that birds would switch to the other feeder and find a reward. Despite this possibility, many birds continued to make side errors and appeared stuck on a specific side, which we referred to as a side bias. This could reflect a habit. Habits involve a specific sequence of repetitive motor behavior and develop outside of awareness (43, 44). While goal-directed learning is rapidly acquired and regulated by its outcome, habit learning is reflexive, elicited by antecedent stimuli rather than its consequences. Habits can occur over the course of days or years and become remarkably fixed (42). Animals with high anxiety show a bias toward habit learning (42). Consistent findings on side bias in fearful hens, here and previously (21), could indicate that fearful animals are sensitive to form habits.

In general, there were no differences in time to make a choice between FC and NFC birds. FC birds only had a longer latency to choose the L reward within a session when sessions included an L trial for the first time. When established behavior responses to the S cue were no longer valid, FC birds were more affected than NFC birds as shown by a longer latency to choose S rewards. Fearful birds might have an increased sensitivity to environmental changes and respond more strongly to changes in cues. This is supported by the longer latency to choose compared to NFC birds for the 75% gray ambiguous cue—the cue most closely resembling the 100% black cue. Fearful birds may be more sensitive to negative outcome of an incorrect choice, i.e., finding no reward while expecting it. Sensitivity to loss, also known as reward sensitivity, has been suggested as a symptom of high anxiety (45). This could explain our results on JB and learning difficulty in FC birds, but further testing is needed to validate this assumption.

Contrary to our hypothesis, more FC birds showed an optimistic-like response to the 25% ambiguous cue compared to NFC birds. While anxious individuals have been shown to display pessimistic behavior (46, 47), similar counterintuitive results have been found (48–50). We are cautious to generalize our conclusions on JB here. The setup of our JB test, i.e., using unrewarded ambiguous cues, could have influenced choice (14). Furthermore, our data can be skewed toward animals which eventually learned the task (5).

We noticed that many birds switched in the final ambiguous trial—with FC birds from S to L and NFC birds from L to S. Birds’ choices could be affected by ambiguous cues being unrewarded. Finding no reward when expecting it can cause frustration and stress which may enhance learning about ambiguous cues being unrewarded. It has been postulated that stressed animals learn these negative associations faster than non-stressed animals (16). This could facilitate a win-stay/lose-shift or win-shift/lose-stay strategy in future ambiguous trials (51). Furthermore, longer latencies to approach ambiguous cues have been postulated to reflect a loss of ambiguity (14). In support of this suggestion, the choice and latency in the final ambiguous trial (25% black) were related to the choice in the 75% black ambiguous trial. Furthermore, FC birds had a longer latency in the 75% black trial and switched more than NFC birds. Partially or fully rewarding ambiguous trials and using a single ambiguous cue could be a way to overcome these potential confounding effects (14). JB can be used to assess emotional valence of birds creating more understanding of what it means to have a fearful predisposition and how it can affect an animal’s welfare.

Our study shows that FC chickens have more difficulty in combining sources of information (cue color, reward size, and location) than NFC chickens. We showed that FC chickens tended to use and stick to a stimulus–response strategy more strongly than NFC chickens. Our results suggest that differences in learning can derive from an animal’s fearful predisposition.

## Ethics Statement

This experiment was approved by the Animal Care and Use Committee of Wageningen University & Research (the Netherlands) in accordance with Dutch legislation on the treatment of experimental animals the ETS123 (Council of Europe 1985) and the 86/609/EEC Directive.

## Author Contributions

EH: design experiment, conduct experiment, data analysis, and writing. CL and BR: help with experiment and writing.

## Conflict of Interest Statement

The authors declare that the research was conducted in the absence of any commercial or financial relationships that could be construed as a potential conflict of interest.

## Abbreviations

FC, fearful characterized; NFC, non-fearful characterized; L, large reward trails; S, small reward trails; NET, novel environment test; OF, open field test; TI, tonic immobility.
